# Climate Change and Mental Health: A Human Rights Perspective

**DOI:** 10.1017/jme.2025.10114

**Published:** 2025

**Authors:** Samvel Varvastian

**Affiliations:** School of Law and Politics, Cardiff University, Cardiff, United Kingdom

**Keywords:** right to health, courts, international law, climate anxiety, children

## Abstract

Climate change-related environmental harms have been observed to negatively affect mental health. While policymakers and courts around the world widely recognise the impacts of climate change on physical heath as potentially endangering human rights, the implications of climate change for mental health have received significantly less attention. This paper analyzed five cases that challenged national response to climate change and the resulting impacts on mental health before four different international human rights protection bodies. Four out of these five cases were dismissed either because the petitioners did not seek prior action before the national authorities, or because their claims were deemed unsubstantiated. Despite these outcomes, the protection bodies’ treatment of these petitions as well as various other ongoing developments show that the human rights approach to climate change and mental health is gradually emerging at the international and domestic levels, but it is still in its early days and there are various challenges to it.

## Introduction

Natural environment is one of the determinants of mental health.^1^ Direct exposure to climate change-related extremes has been observed to negatively affect mental health: heatwaves, wildfires, drought, and storms can lead to diagnosable mental health disorders, including post-traumatic stress disorder (PTSD), depression, and even suicide.^2^ For example, a 1°C increase in average monthly temperature in the US over the years 1968–2004 was associated with increased monthly suicide rate by 0.68%, while the same temperature increase in Mexico over the years 1990–2010 was associated with increased monthly suicide rate by 2.1%.^3^ A systematic review of published studies on mental health effects of high ambient temperatures and heat waves in other countries, including Australia, Belgium, Canada, Japan, Kazakhstan, Korea, and the UK, also found a positive association between increasing temperature and suicide frequency.^4^ Similarly, US cities that were exposed to Hurricane Katrina had a 4% increase in occurrence of mental health issues compared with unaffected areas.^5^ Awareness about climate change and related environmental harms can also lead to anxiety.^6^ For example, of the 10,000 children from ten countries who took part in a survey in 2021, nearly 60% reported that they were very worried or even extremely worried about climate change.^7^

Climate change also feeds into global-scale pollution and biodiversity loss, thus forming part of the triple planetary crisis,^8^ and resulting in additional impacts, including on mental health.^9^ These negative impacts are expected to become more common and severe in the future,^10^ while vulnerable populations, including people with preexisting mental health disorders, Indigenous communities, women, and so forth, will remain disproportionately affected by these impacts.^11^ The severity of these impacts may result in human rights violations.^12^

While the impacts of climate change on physical heath are globally recognized as potentially endangering human rights,^13^ the implications of climate change for mental health from a human rights perspective have received significantly less attention and have not been thoroughly explored in either policy or research. And yet, concerns about the potential implications of climate change for mental health were raised as early as 1990 by the Intergovernmental Panel on Climate Change (IPCC) in its first assessment report,^14^ and subsequently expanded in its later reports.^15^ This lack of attention reflects various underlying challenges to the human rights approach to mental health identified by the United Nations (UN) Special Rapporteur on the right to health,^16^ including the longstanding practice of sidelining mental health, the focus on treatment rather than prevention,^17^ and the systemic failure to consider the social determinants of mental health.^18^ In light of these challenges, the Special Rapporteur on the right to health has called for a rights-based approach to climate change mitigation and adaptation “with mental health at its heart.”^19^

Indeed, the existing provisions in various international human rights treaties already provide at least some grounds for such an approach. For example, while not explicitly referred to, the obligation to protect mental health, including in the context of environmental degradation, falls within the scope of the right to respect for private and family life under Article 8 of the European Convention on Human Rights (ECHR).^20^ The European Court of Human Rights (ECtHR) has long interpreted the concept of “private life” as encompassing, among other things, physical and psychological integrity of a person.^21^ Accordingly, the ECtHR has held on many occasions that depending on their severity, the impacts of environmental harms on mental health can indeed result in violation of Article 8.^22^ Similarly, the UN Human Rights Committee (HRC) has interpreted the right to private and family life and home under Article 17 of the International Covenant on Civil and Political Rights (ICCPR) as potentially endangered when the adverse consequences of environmental degradation are serious because of its intensity or duration and the physical or mental harm that it does.^23^

Still, all these cases have usually focused on traditional environmental harms such as air, water, and soil pollution, and noise, and it was not until recently when human rights bodies had an opportunity to interpret these provisions in the context of climate change.

The goal of this paper was to explore whether the existing international human rights instruments can be used to seek protection of mental health from the impacts of climate change. To that end, this paper aimed to answer the following questions: (1) What impacts of climate change on mental health did petitioners allegedly experience and what was the national response to climate change that they complained about? (2) Which provisions of human rights treaties did petitioners invoke in relation to their complaints? (3) How do international human rights courts and treaty bodies interpret these provisions in the context of climate change? (4) Do the outcomes of these cases reflect any broader trends and do they indicate any opportunities for or challenges to the rights-based approach to climate change and mental health?

## Climate change and mental health: the evolution of human rights cases

Historically, the Inter-American Commission on Human Rights (IAComHR) had an opportunity to develop a rights-based approach to climate change and mental health well before any other international human rights court or treaty body. In 2005, the Chair of the Inuit Circumpolar Conference, Sheila Watt-Cloutier, brought a petition before the IAComHR on behalf of herself, a group of other individuals, and all Inuit of the Arctic regions of the US and Canada against the US as then the world’s largest emitter of greenhouse gases (GHGs).^24^ The petitioner challenged the lack of federal GHG emissions reduction targets and gaps in regulatory response to major emissions sources such as power plants and vehicles, as well as the lack of participation in international efforts to reduce GHG emissions. This lack of federal action allegedly violated the right to health under Article XI of the American Declaration of the Rights and Duties of Man and various other rights because of the wide range of impacts of climate change on Inuit life, culture, and physical and mental health.^25^ With regard to mental health, the petition highlighted psychological stresses due to the Inuit’s reduced opportunities for subsistence hunting, fishing, herding, and gathering, their growing anxiety due to unfamiliarity and unpredictability of changing weather patterns and associated dangers related to travel, as well as due to destruction and relocation of their homes and communities. In 2006, the IAComHR dismissed the petition as not providing the necessary information to determine whether the alleged facts would amount to a violation of rights protected by the American Declaration.^26^

For many years, the Inuit petition remained the only opportunity for the development of the rights-based approach to climate change and mental health at the international level, until a new opportunity arose with the submission of several climate change-focused petitions to other international treaty bodies in the late 2010s and early 2020s. Among these were petitions brought by children against the governments of Argentina, Brazil, France, Germany, and Turkey before the UN Committee on the Rights of the Child (CRC-Com).^27^ The petitioners challenged the lack of sufficiently ambitious GHG emissions reduction targets by the defendants that would be consistent with the Paris Agreement’s goal to keep global warming well below 2°C above pre-industrial levels and pursue efforts to limit it to 1.5°C. Accordingly, the petitioners alleged that the defendants violated the right to the highest attainable standard of health under Article 24 of the UN Convention on the Rights of the Child (CRC)^28^ and various other rights by failing to properly address climate change mitigation and adaptation, which, among other things, affected children’s mental health and led to climate anxiety. The petitioners referred to a range of particular mental health impacts, including developing depression after learning about climate change in school, psychological trauma after experiencing wildfires, feelings of powerlessness and despair when thinking about the future, and so forth. The CRC-Com dismissed all these petitions because the petitioners did not seek prior action before the national authorities.^29^ Nevertheless, the CRC-Com held that despite the global nature of climate change, the resulting global environmental damage, the universal impacts on human rights, and the necessity to deal with this problem at the global level, states still have individual responsibility for their own acts or omissions in relation to climate change and their contribution to it.^30^ The CRC-Com was also persuaded that petitioners were sufficiently affected to claim protection of their CRC rights, including from the impacts of climate change on their mental health.

One year later, an even more notable outcome occurred in the HRC case of *Daniel Billy v Australia*, which concerned the alleged failure by the Australian government to take mitigation and adaptation measures against the impacts of climate change on an Indigenous minority group of the Torres Strait islands.^31^ Specifically, the petitioners challenged the lack of implementation of an adaptation program to ensure the long-term habitability of the islands despite the ever-growing threats that are primarily related to sea level rise, including flooding and erosion, and the resulting disruption to food production and traditional ways of life. Furthermore, the petitioners alleged the government’s failure to mitigate the impacts of climate change, as evidenced by the fact that Australia’s GHG emissions have increased by over 30% in the last three decades, making the country the second largest per capita emitter in the world in 2017. The HRC considered the impacts of climate change on physical and mental health — including “environmental degradation on Indigenous lands in communities where subsistence is highly dependent on available natural resources and where alternative means of subsistence and humanitarian aid are unavailable” — stemming from the delay in sea wall construction and the lack of other necessary national measures to be severe enough to violate Article 17 of the ICCPR.

In contrast, the ECtHR did not seize the first substantial opportunity to interpret Article 8 of the ECHR in the context of climate change and mental health in the case of *Duarte Agostinho v Portugal*,^32^ which was the first of several climate change-focused petitions submitted to the ECtHR in the early 2020s.^33^ *Duarte Agostinho* was filed by a group of Portuguese children against 33 European states; the petitioners complained about the release of GHG emissions within the defendant-states’ national territory and offshore areas, the permission of export of fossil fuels that are extracted in these countries and the import of goods that result in GHG emissions, as well as the permission for corporate entities within their jurisdiction to extract fossil fuels and contribute to emissions overseas. This contribution to climate change arguably violated various ECHR rights, including the right to respect for private life under Article 8. Unlike other climate change petitions to the ECtHR, the petition in *Duarte Agostinho* had a significant focus on mental health: for example, the children-petitioners complained about experiencing anxiety and horror during the 2017 wildfires in Portugal, driven by climate change-induced heatwaves, without suffering any physical harm. In particular, the petitioners claimed that they were horrified to know that the wildfires were causing death and destruction close to their home; they were also increasingly worried and upset about the future impacts of climate change. The Grand Chamber of the ECtHR dismissed the petition because petitioners did not seek action before national authorities first, even though there were available legal instruments for such action, namely, the 2017 Portuguese law that allowed victims of wildfires — including those who experienced mental health harms — to seek compensation. Claims against states other than Portugal were also dismissed.

Apart from dismissing the petition in *Duarte Agostinho*, the ECtHR also dismissed another petition — *Damien Carême v France* — that also raised the issue of climate change and mental health, albeit to a much lesser extent: the petitioner complained that the alleged insufficiency of national action to address the impacts of climate change and the failure to take all necessary measures to curb GHG emissions prevented him “from serenely envisaging himself in his home in the future,” thus affecting his right to private and family life under Article 8 of the ECHR.^34^ But the Grand Chamber was unconvinced and held that since “almost anyone could have a legitimate reason to feel some form of anxiety linked to the risks of the adverse effects of climate change in the future,” allowing petitions where there is no “pressing need to ensure an applicant’s individual protection from the harm which the effects of climate change may have on the enjoyment of their human rights” would undermine the protection system under the ECHR.

## Emerging rights-based approach to mental health?

At first glimpse, the analyzed cases present a sharp contrast between the approaches taken by the UN human rights protection bodies (HRC and CRC-Com) and the regional bodies (ECHR and IAComHR). However, this contrast may be less pronounced considering the broader context in which the respective cases have developed. For example, the dismissal of the Inuit petition by the IAComHR took place around 10 years before the emergence of systematic development of the rights-based approach to climate change both at the regional level and in the US itself.^35^ It is therefore far from obvious that such a petition would be dismissed in a similar way nowadays. In fact, in 2021, together with the Office of the Special Rapporteur on Economic, Social, Cultural, and Environmental Rights (REDESCA), the IAComHR adopted a resolution on the scope of Inter-American human rights obligations in climate emergency, in which it emphasized that “climate change is one of the greatest threats to the full enjoyment and exercise of human rights of present and future generations, to the health of ecosystems and all species that inhabit the planet.”^36^ In this resolution, the IAComHR outlined human rights obligations of states to adopt and implement policies and other measures to address climate change and environmental degradation, ensure protection of vulnerable populations, and so forth, as well as responsibilities of businesses.

Another example is the recent interpretation of Article 8 of the ECHR by the ECtHR. On the very same day that the ECtHR Grand Chamber dismissed the petitions in *Duarte Agostinho* and *Carême*, it held in another case — *Klimaseniorinnen v Switzerland* — that the defendant state had failed to protect the right to respect for private and family life under Article 8 of the ECHR because of “some critical lacunae” in the Swiss authorities’ process of establishing the relevant national regulatory framework for climate change as well as because of their failure to meet the state’s GHG emissions reduction targets.^37^ The ECtHR decision in *Klimaseniorinnen* thus marked the first success for petitioners in climate change litigation before an international court, adding to a line of similar decisions in cases before domestic courts in Europe and beyond,^38^ and becoming a potential catalyst for further rights-based action on climate change.^39^ Therefore, the dismissal of claims related to the impacts of climate change on mental health in *Duarte Agostinho* and *Carême* should not be viewed as barring the potential application of Article 8 of the ECHR in similar claims in the future.

The same is true with respect to potential future petitions to the CRC-Com, particularly, following the adoption of the General Comment No. 26 on children’s rights and the environment, with a special focus on climate change by the CRC-Com in May 2023.^40^ In that General Comment, among other things, the CRC-Com identified “children’s current and anticipated psychosocial and mental health conditions caused by environmental harm, including climate change-related events” as an area of concern for the realization of the right to the highest attainable standard of health under Article 24 of the CRC. The CRC-Com was also convinced that there is a “clear emerging link between environmental harm and children’s mental health, such as depression and eco-anxiety” and that it “requires pressing attention, both in terms of response and prevention programmes, by public health and education authorities.” The CRC-Com also recognized the interrelated nature of the protection of mental health and realization of various other children’s rights. Accordingly, the CRC-Com outlined a list of measures that states should immediately take to protect children’s rights, including reducing both outdoor and indoor air pollution, phasing out the use of fossil fuels and investing in renewable energy, banning the introduction of polluting substances into the marine environment, and so forth.

Last, but certainly not least, the development of the rights-based approach to climate change and mental health is also taking place at the national level. For instance, as *Duarte Agostinho* shows, there are national laws that allow persons whose mental health has been affected by climate change-related environmental harms to seek justice. In *Duarte Agostinho*, it was the law that allowed victims of wildfires to seek compensation for mental health harms. Such laws are consistent with the recognition of the right to health in over 100 constitutions around the world^41^ and in international law, including Article 12 of the International Covenant on Economic, Social and Cultural Rights that recognizes “the right of everyone to the enjoyment of the highest attainable standard of physical and mental health.”^42^ The obligations to protect and fulfill the right to health under Article 12 extend to various measures against environmental health harms, including adoption and enforcement of laws concerning environmental pollution, biodiversity loss, and so forth.^43^ Such laws are also consistent with the right to a clean, healthy and sustainable environment, that also enjoys global recognition in national and international law.^44^ Finally, it is also notable that domestic courts in countries like the US^45^ and Ireland^46^ have considered the impacts of climate change on mental health as potentially endangering constitutionally-protected rights.

## Persisting challenges

That said, the human rights approach to climate change and mental health is still in its very early days; therefore, it is still extremely fragmented and incomplete. For instance, the position of the CRC-Com on climate change and mental health concerns only the rights of children and does not extend to various other vulnerable populations, whereas other human rights courts and treaty bodies have not even addressed the matter. For instance, it remains to be seen what approach the Inter-American Court of Human Rights (IACtHR) takes with respect to the impacts of climate change on mental health in its forthcoming advisory opinion on the scope of state obligations to address climate change.^47^ The forthcoming advisory opinion is a historic opportunity for the IACtHR to build on its 2017 advisory opinion that addressed the link between the environment and human rights.^48^ In the latter opinion, the IACtHR recognized the adverse effects of climate change and outlined the obligations of states to avoid transboundary environmental damage that could affect the rights of persons outside their territory. While on that occasion the IACtHR did not directly discuss the impacts on mental health, it referred to relevant standards under Article 5 of the American Convention on Human Rights (ACHR) (right to physical and mental integrity),^49^ which can be endangered by environmental harms, as well as Article 8 of the ECHR.

For its part, the African Commission on Human and Peoples’ Rights (AfCHPR) has a similar historic opportunity to interpret the relationship between climate change and mental health through the lens of Articles 22 (right of peoples to economic, social and cultural development) and 24 (right of peoples to a satisfactory environment favorable to their development) of the African Charter on Human and Peoples’ Rights (ACHPR)^50^ in its ongoing study on the impacts of climate change on human rights in Africa.^51^ These provisions of the ACHPR have already been applied in the context of traditional environmental harms by the African Court on Human and Peoples’ Rights that held the defendant-states responsible for failure to prohibit and tackle environmentally-harmful practices such as dumping of hazardous waste.^52^ Whether the AfCHPR interprets these articles with respect to the impacts of climate change on mental health remains to be seen, but it is notable that in its 2009 resolution that originally authorized the ongoing study, the AfCHPR made no reference to mental health, despite expressing concern about the impacts of climate change on physical health.^53^

Furthermore, not a single court or treaty body has thus far considered the impacts of climate change on mental health in detail. As a result, the emerging human rights approach to climate change and mental health lacks any in depth and systematic consideration of the best available science. A good example of this is climate anxiety — a condition when an individual’s worry about climate change begins to affect their life.^54^ Climate anxiety is currently not listed in psychiatric and medical classification systems such as DSM-5^55^ and ICD-11.^56^ The IPCC 6^th^ Assessment Report identified climate anxiety as one of the areas where more evidence about the prevalence and severity of this condition is needed, but the results of the existing surveys in many countries generally point towards positive correlation between the perceived threat of climate change and lower mental health.^57^ These findings confirm the urgent need to assess the full scale of climate change impacts on mental health from a human rights perspective in order to protect such fundamental interests as life, health, and wellbeing.

And yet, the use of the best available science in the existing rights-based action has been rather limited. For instance, the lead author of the survey on climate anxiety among children mentioned earlier^58^ produced an expert report on the condition of the petitioners in *Duarte Agostinho* as evidence. The report concluded that the petitioners suffered from an “‘Adverse Childhood Experience’ linked to prolonged climate anxiety” as well as from “a form of mental suffering called ‘moral injury’ caused by their awareness of the failure of those in authority to protect them.” The findings of this report were consistent with the findings of the survey itself, because the latter also revealed high rate of concern about climate change and greater feelings of betrayal associated with perceived inadequate governmental response to climate change among the surveyed children, although it is notable that the survey did not measure the actual severity of climate anxiety by any psychological scale.^59^

Nevertheless, the evidentiary value of the report on the petitioners’ mental health was challenged by some of the defendant-states. In particular, the defendants stated that there was no evidence of the petitioners being examined by an independent expert “qualified to carry out medico-legal examinations,” and no evidence of the petitioners being admitted to a hospital or treated either for anxiety and depression generally, or for climate anxiety specifically.^60^ That said, since the construct of climate anxiety is new and complex, with no standardized measures to assess the severity of its impacts on mental health or even a universally accepted definition,^61^ it is far from clear whether any such evidence could be provided at this point in time.

In fact, the ECtHR observed that there was “a significant lack of clarity as regards the applicants’ individual situations, which makes it difficult to examine whether they satisfy the victim-status criteria.”^62^ The latter criteria were set out in *Klimaseniorinnen*, and required the applicants to show that they were personally and directly affected by the alleged failures of the state to combat climate change. More specifically, the applicant would need to demonstrate the following circumstances: (a) the applicant being “subject to a high intensity of exposure to the adverse effects of climate change,” or in other words, that “the level and severity of (the risk of) adverse consequences of governmental action or inaction affecting the applicant must be significant;” and (b) “there must be a pressing need to ensure the applicant’s individual protection, owing to the absence or inadequacy of any reasonable measures to reduce harm.”^63^ The ECtHR stressed that the threshold for fulfilling these criteria is “especially high,” and that a wide range of factors need to be considered as part of the victim status assessment, including, but not limited to, “prevailing local conditions and individual specificities and vulnerabilities,” “the actuality/remoteness and/or probability of the adverse effects of climate change in time,” “the specific impact on the applicant’s life, health or well-being,” “the magnitude and duration of the harmful effects,” and “the scope of the risk (localized or general).”^64^

In *Duarte Agostinho*, the ECtHR considered that the lack of clarity with regard to the petitioners’ individual situations could be explained, in particular, by the fact that the petitioners did not seek action before national authorities first^65^ — a process that would have likely resulted in more extensive evidence than the one that was actually available. But in any case, because the ECtHR dismissed the petition without assessing the actual impacts on the children-petitioners, the potential use of the petitioners’ produced evidence, or indeed, of the supporting research on climate anxiety among children in rights-based action remains unknown.

In contrast, the CRC-Com considered the scientific evidence on the impacts of climate change on children’s health, including mental health impacts such as climate anxiety, depression, PTSD, and so forth, submitted by the petitioners in *Sacchi*,^66^ which likely explains the Committee’s recognition of the fact that the petitioners were sufficiently affected to claim protection of their rights.

Similarly, scientific evidence on the impacts of climate change on mental health was considered by domestic courts in the US case of *Held v Montana*, when a group of US children-petitioners successfully challenged specific provisions of Montana’s environmental and energy policy legislation that forbade the state regulatory bodies from considering the impacts of GHG emissions or climate change in their environmental impact assessment.^67^ The children claimed that the impacts of climate change, including on their mental health — such as traumatic experience during extreme weather events, despair and fear because of the loss of their cultural connection to the land, and so forth — violated their right to a healthy environment under Articles II and IX of the Montana’s Constitution. The first instance court heard testimony from several qualified experts, including a board-certified general and forensics clinical psychiatrist and a board-certified pediatrician, on the psychological harms caused by climate change to the claimants, as well as on the findings of the IPCC. The court held that although “mental health injuries directly resulting from State inaction or counterproductive action on climate change” are not on their own actionable, “mental health injuries stemming from the effects of climate change on Montana’s environment, feelings like loss, despair, and anxiety” are indeed actionable. The court also denied the defendant’s motion for a psychiatric examination of the children-claimants, as it considered that mental health was not genuinely in controversy and that the children’s claim was actionable even without considering their psychological harms. Overall, even though the claimants were successful, including when the state appealed to the Supreme Court of the State of Montana,^68^ the courts’ consideration of the impacts of climate change on mental health in this case was very limited.

Finally, because of its emerging nature, the rights-based approach to climate change and mental health is still very far from incorporating consideration of the intertwined nature of climate change and other elements of the triple planetary crisis. And yet, such considerations are already common in rights-based approaches to climate change and physical health, and they reflect the recognition of the fact that climate change is driven by the very processes that cause global pollution of air, water, and land, and biodiversity loss.^69^ These considerations would also contribute to greater awareness of the traditionally overlooked social determinants of mental health that are vital for ensuring the rights-based approach.

In other words, the actual relationship between climate change and mental health in the existing rights-based cases — both at the international and domestic level — has so far remained largely outside the courts’ and treaty bodies’ scrutiny, although there seems to be a general recognition of the fact that the impacts of climate change on mental health can potentially result in human rights violations. This recognition reiterates the importance of concerns raised by the Special Rapporteur on the right to health.^70^ It also confirms the need for coordinated and collaborative policy measures on climate change mitigation and adaptation to address the implications for mental health, and the need for greater involvement of mental health practitioners to support psychosocial wellbeing within a changing climate, as well as the need for further research to better understand the linkages between climate change and mental health.^71^

As long as the existing challenges to the rights-based approach to climate change and mental health remain unaddressed, they will undermine the efficacy of the existing international human rights instruments and render them of little use in terms of ensuring proper protection of those whose mental health is endangered by one of the greatest challenges of our time. Therefore, these challenges require a coherent and science-informed response. As the rights-based approach to climate change and mental health is still in its early days, policymakers and courts have a historic opportunity to adopt relevant practices and standards that will form the basis of such a response.

## Conclusion

There is a global recognition of the fact that the impacts of climate change on human health can result in human rights violations. But these concerns have been predominantly raised in the context of physical health, while the implications of climate change for mental health from a human rights perspective have received overwhelmingly less attention. Still, the rights-based approach to climate change and mental health already exists at the international and domestic levels, albeit it is still in its very early days, and it faces various challenges. Among the most prominent such challenges are fragmentation, systematic neglect of mental health as an issue of concern, and lack of consideration of the best available scientific evidence on the link between climate change, other planetary crises, and mental health. As long as these challenges remain unaddressed, the existing international human rights instruments are likely to fall short of ensuring a proper level of protection of those whose mental health is endangered by one of the greatest challenges of our time.
